# Clinical Application of rhBMP-2 and Three-Dimensinal Preformed Titanium Mesh with Allograft and Xenograft for Peri-Implant Horizontal and Vertical Bone Augmentation–A Narrative Review with Technical Report

**DOI:** 10.3390/jcm14134788

**Published:** 2025-07-07

**Authors:** Yeong Wook Kim, Saverio Cosola, Young Sam Kim, Young Min Park, Ugo Covani, Aimone Fabbri, Giovanni Battista Menchini-Fabris

**Affiliations:** 1 Wonju Dental Clinic, Wonju 26437, Republic of Korea; wonjudental@naver.com; 2 Department of Stomatology, Tuscan Stomatologic Institute, Foundation for Dental Clinic, Research and Continuing Education, 55041 Camaiore, Italy; covani@covani.it (U.C.);; 3 Department of Dentistry, Unicamillus—Saint Camillus International University of Health and Medical Sciences, 00100 Rome, Italy; 4 Gangam Dental Office, Seoul 06614, Republic of Korea; doctorkimys@gmail.com (Y.S.K.);; 5 San Rossore Dental Unit, Viale delle Cascine 152, San Rossore, 56122 Pisa, Italy; 6 Department of Psychology and Health Sciences, Pegaso University, Business District, Isola F2, 80143 Napoli, Italy

**Keywords:** human bone morphogenetic protein-2, 3D preformed titanium mesh, bone reconstruction, dental implants, bone substitute, bone bio-materials, osteoinduction

## Abstract

The reconstruction of a severely resorbed alveolar bone is a significant challenge in dental implantology and maxillofacial surgery. Traditional bone grafting materials, including autogenous, allogeneic, xenogeneic, and alloplastic materials, have limitations such as donor site morbidity, limited availability, and prolonged maturation periods. To address these challenges, recombinant human bone morphogenetic protein-2 (rhBMP-2) has emerged as a potent osteoinductive factor that facilitates bone regeneration without the need for additional donor site surgery. This study introduces a box technique which combines rhBMP-2 (CowellBMP^®^, Cowellmedi, Busan, Republic of Korea) with a 3D-preformed titanium mesh (3D-PFTM), utilizing a mixture of allografts and xenografts for horizontal and vertical alveolar ridge augmentation. The technique leverages the structural stability provided by the OssBuilder^®^ (Osstem, Seoul, Republic of Korea), a preformed titanium mesh, that allows for simultaneous implant placement and vertical ridge augmentation. This technique not only reduces the treatment time compared to traditional methods but also minimizes post-operative discomfort by eliminating the need for autogenous bone harvesting. Clinical outcomes from this technique demonstrate successful bone regeneration within a shorter period than previously reported techniques, with excellent bone quality and implant stability being observed just four months after vertical augmentation. In conclusion, the so called BOXAM (BMP-2, Oss-builder, Xenograft, Allograft, Maintenance) technique presents a promising therapeutic strategy for alveolar bone reconstruction, particularly in cases of severe bone resorption. Further studies are needed to evaluate the long-term outcomes and potential limitations of this approach, especially in scenarios where the inferior alveolar nerve proximity poses challenges for fixture placement.

## 1. Introduction

The reconstruction of a severely resorbed alveolar bone poses one of the greatest challenges in dental implantology and maxillofacial surgery. This reduction in bone volume compromises the stability and long-term success of implants, creating significant challenges for both patients and clinicians. Alveolar bone resorption is a natural physiological process that occurs after tooth extraction and leads to a decrease in the height and width of the alveolar ridge over time, potentially rendering the site inadequate for implant placement [1].

To address these challenges, various bone grafting materials have been developed such as autogenous bone harvested from the patient, allogeneic bone, xenogenic bone, and alloplastic materials.

Autogenous bone is considered the ideal and standard graft material for bone grafting because it possesses osteogenic, osteoinductive, and osteoconductive properties, which enable it to promote the most effective bone regeneration [2].

However, harvesting autogenous bone requires an additional surgical procedure at another site, which can increase patient discomfort after surgery, extend the recovery period, and raise the risk of complications. Additionally, autogenous bone grafts have limitations, such as the limited amount of bone that can be harvested and a high resorption rate after grafting. To overcome these drawbacks, various bone substitutes have been developed, and their clinical application and effectiveness have been widely studied. However, these bone substitutes often need a long maturation period after grafting to achieve sufficient bone quality for implant placement [3,4].

Further, allogeneic bone, xenogenic bone, and alloplastic materials offer the advantages of not requiring harvesting from the patient’s own body, which thereby eliminates patient discomfort and provides an unlimited supply of materials for grafting. However, they have lower osteogenic potential compared to autogenous bone and are associated with higher costs [5].

With the potential to overcome the limitations of traditional bone graft materials, recombinant human bone morphogenetic protein-2 (rhBMP-2) has emerged as a particularly promising option. This is a potent osteoinductive factor that demonstrates significant effectiveness in promoting bone regeneration across various clinical applications [6]. Unlike autogenous bone grafts, rhBMP-2 can achieve excellent bone formation without the need for additional donor site surgery, and its use thus avoids the complications associated with autograft harvesting [7]. The bone morphogenetic protein (BMP) is an important member of the transforming growth factor-beta (TGF-β) superfamily, a group of highly conserved homologous signaling proteins that play an important role in embryogenesis, organogenesis, cell proliferation, and stem-cell differentiation [8].

The discovery of BMP marked a significant breakthrough in bone regenerative medicine. It was first identified in 1965 by Marshall Urist, who demonstrated that demineralized bone matrix could induce bone formation when implanted in non-osseous tissue [9]. This discovery laid the groundwork for understanding how bone forms in response to injury, leading to a surge in BMP research.

Wozney and colleagues identified and characterized several BMP subgroups, including BMP-2, BMP-4, and BMP-7, elucidating their specific biological functions. These studies were pivotal in explaining how growth factors such as BMPs induce bone formation through cellular signaling pathways [10]. To date, approximately 20 BMP family members have been identified and characterized. BMP is a dimeric molecule composed of two polypeptide chains that are linked by a single disulfide bond. According to the structural similarity of BMP amino acid sequences, the BMP family members are generally divided into four categories: BMP-2/-4; BMP-5/-6/-7/-8; BMP-9/10; and BMP-12/-13/-14 [11]. BMP-2, in particular, has emerged as a powerful osteoinductive agent, and is widely used in both dental and orthopedic surgery [12,13].

The reconstruction of alveolar bone in the implant area is a critical aspect of oral implantology. Several clinical methods are available for addressing alveolar bone defects, including guided bone regeneration (GBR), onlay bone grafting, bone extrusion, bone splitting, and distraction osteogenesis. Among these, GBR stands out as one of the most commonly employed techniques due to its straightforward procedure, low technical sensitivity, stable osteogenesis, and capacity for multidirectional bone formation [14]. In the GBR process, barrier membranes are used to maintain space and prevent epithelial cell migration. These membranes are categorized into absorbable and non-resorbable types based on their resorption characteristics. Resorbable membranes have the advantage of not requiring a second surgery for removal, but many studies have reported that their effectiveness in cell occlusion and space maintenance is inferior to that of non-resorbable membranes. On the other hand, non-resorbable membranes provide excellent space maintenance and can be used in areas with severely resorbed alveolar bone [15].

The most widely used non-resorbable membrane is the polytetrafluoroethylene (PTFE) membrane. However, in cases where there is insufficient bony support around the defect, it can be challenging for the non-resorbable membrane to maintain its shape due to excessive pressure from the flap. To address this issue, fixation using screws and pins has been employed. Despite this, shape deformation of the membrane can still occur, which has lead to research into alternative solutions [16,17].

Titanium mesh provides structural stability during the reconstruction of highly resorbed alveolar ridges, protecting the bone graft material and maintaining the necessary space for new bone formation [18]. The non-resorbable nature of titanium mesh prevents the collapse or migration of the regenerating bone, supporting stable long-term outcomes [14].

Titanium mesh is particularly advantageous in vertical and horizontal ridge augmentation. In vertical ridge augmentation, it increases the height of the alveolar ridge, facilitating implant placement, while in horizontal ridge augmentation, it widens the ridge to ensure implant stability, although some drawbacks and complications have been reported [19]. The mesh typically comes in a rectangular shape and requires careful trimming and bending to match the defect’s morphology, which necessitates significant clinical skill, expertise, and time. Additionally, the modeling process may produce irregular and sharp edges, which can expose soft tissues to mechanical trauma, potentially resulting in flap perforation and mesh exposure. Such complications may lead to infection and subsequent partial or complete loss of the initial bone augmentation. To address these disadvantages, laser sintering has been suggested as a method to create customized titanium meshes using computer-aided design/computer-aided manufacturing (CAD/CAM) technology [20]. However, this method may require various types of specialized equipment and incur significant costs, which makes it likely to be used only in a limited capacity.

Further, to overcome the disadvantages of conventional Ti-mesh, a three-dimensional preformed Ti mesh (3D-PFTM) was developed. This 3D-PFTM enhances the prognosis of alveolar ridge augmentation and is user-friendly, as it requires no cutting or bending. This design also shortens the surgery time and lowers the mesh exposure rate by eliminating sharp edges. Moreover, it has been noted that minimizing the invasion of the bone graft site during the second surgery is beneficial, allowing GBR to be performed simultaneously with implant placement for bony defects around the implant [21]. Choi et al. reported that 3D-PFTM should be considered a valuable option for GBR in the treatment of peri-implant non-contained horizontal defects [22].

Recent studies have demonstrated the efficacy of combining 3D-PFTM with rhBMP-2 in complex reconstruction scenarios. This combination improves the reliability of bone regeneration and yields better outcomes in complex alveolar defects [23]. However, there have been few reports of successful bone regeneration using 3D-PFTM in combination with rhBMP-2.

This study aims to present the clinical efficacy of using 3D-PFTM in conjunction with rhBMP-2 for the reconstruction of severely resorbed alveolar bone, both horizontally and vertically. Through a narrative review with case reports, it seeks to introduce a new paradigm in maxillofacial reconstruction with this approach.

## 2. Materials and Methods

These case reports include patients who visited Wonju Dental Clinic (Wonju, Republic of Korea) and had non-contained horizontal defects or vertical defects. The cases were selected based on the use of rhBMP-2 (CowellBMP^®^, Cowellmedi, Republic of Korea), 3D-PFTM (Oss-Builder^®^, Osstem, Republic of Korea), freeze-dried particulate bone allograft (Regenoss^®^, SureOss^®^, MyBone^®^), and deproteinized bovine (A-Oss^®^) or porcine bone (THE Graft^®^) for horizontal or vertical alveolar ridge augmentation. All patients were followed up with for more than 18 months. Data were analyzed at Unicamillus International University by two authors who obtained the statics (YSK and SC). The local ethical committee approved the retrospective study with protocol number E00332-2024.

### 2.1. Preparation of Bone Graft Material with rhBMP-2

**Figure 1:**
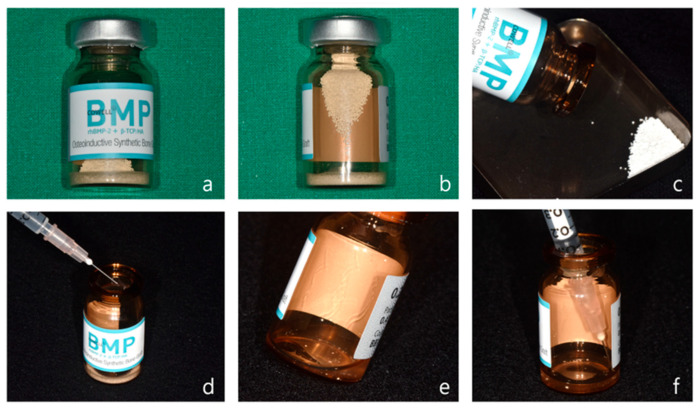
Preparation of rhBMP-2. (**a**) Front view of CowellBMP 0.25 g. (**b**) Synthetic bone and freeze-dried rhBMP-2 powder stored in a glass vial. (**c**) The synthetic bone is removed from the vial after opening the cap. (**d**) A total of 0.25–0.5 cc of sterile water is injected into the vial. (**e**) The vial is gently shaken to dissolve the rhBMP-2 powder in the sterile water. (**f**) The dissolved rhBMP-2 solution is drawn back into a 1 cc syringe.

For horizontal and vertical alveolar ridge augmentation, one vial of CowellBMP^®^ 0.25 g is used per 2–3 implant sites (Figure 1a). Each CowellBMP^®^ vial contains tricalcium phosphate/hydroxyapatite (TCP/HA) graft material along with freeze-dried rhBMP-2 in powder form (Figure 1b). To prepare the solution, carefully open the vial and remove the graft material (Figure 1c). Using a 1 cc syringe, add from 0.25 to 0.5 cc of sterile water to the 0.25 g CowellBMP^®^ vial (Figure 1d). Gently mix to ensure complete dissolution of the rhBMP-2 in the sterile water (Figure 1e,f). The allograft and xenograft materials designated for alveolar ridge augmentation should be carefully separated and prepared. Subsequently, saturate each graft material with the rhBMP-2 solution to ensure optimal efficacy during the augmentation procedure.

### 2.2. Preparation of 3-Dimensional Preformed Titanium Mesh

**Figure 2:**
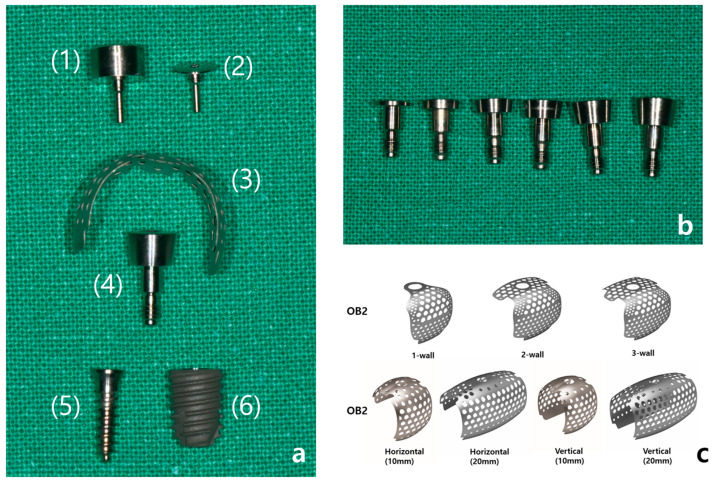
(**a**) Components of the Ossbuilder system: (1) healing cap, (2) cover cap, (3) Ossbuilder titanium mesh, (4) anchor, (5) tenting screw, (6) fixture. (**b**) Ossbuilder anchors of varying heights, from left to right: 0.5, 1.0, 1.5, 2.0, 2.5, 3.0 mm. (**c**) Different types of Ossbuilder Ti-mesh.

OssBuilder (OB) is a prefabricated product made by the company Osstem, and this system consists of an anchor (0–3 mm) that connects to the fixture, a titanium mesh, and a cover cap or healing cap that secures the titanium mesh to the anchor. When immediate fixture placement is not possible, a tenting screw can be used as an alternative to the fixture (Figure 2a). The length of the anchor is selected based on the condition of the alveolar bone surrounding the implant and the depth at which the implant is placed (Figure 2b). To minimize the risk of long-term peri-implant infections and to ensure biological safety, a 3 mm anchor is generally preferred, as it allows for 3–4 mm of soft tissue coverage above the implant.

The titanium mesh is broadly categorized into OB2 and OB3 types, depending on the shape of the alveolar bone defect. The OB2 type is used for reconstructing alveolar bone defects with small vertical or horizontal deficiencies, such as in cases of fenestration or dehiscence, and is available in 1-wall, 2-wall, and 3-wall types based on the defect shape in the buccal, proximal, and lingual areas. The OB3 type is used for more severe alveolar bone recession, being designed to achieve 5–10 mm vertical or horizontal bone augmentation, and is available in horizontal and vertical types according to the desired form of augmentation (Figure 2c). The selected titanium mesh may also require pre-adjustment according to the implant placement site and the shape of the defect.

**Figure 3:**
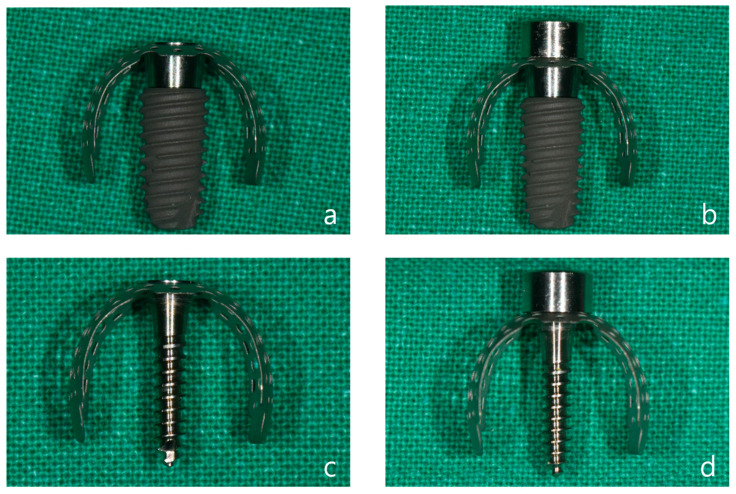
(**a**) A 5.0 × 10 mm fixture with a 3 mm anchor, OB3 vertical type mesh, and cover cap attached. (**b**) A 5.0 × 10 mm fixture with a 3 mm anchor, OB3 vertical type mesh, and healing cap attached. (**c**) An 11.5 mm tenting screw with OB3 vertical type mesh and cover cap attached. (**d**) An 11.5 mm tenting screw with OB3 vertical type mesh and healing cap attached.

In cases where immediate implant placement after extraction results in insufficient soft tissue coverage, where there is an abundance of attached gingiva surrounding the surgical site, or where the bone graft volume is minimal and the site is less influenced by the tongue or occlusal forces, a healing cap may be used (Figure 3a–d). However, based on clinical experience, there have been instances where the healing cap becomes loose despite being tightly secured by hand after the titanium mesh is applied. Additionally, the bone quality achieved with a healing cap tends to be slightly inferior compared to that achieved with a cover cap during the second surgery. Therefore, it is recommended to use a cover cap, particularly in cases requiring significant horizontal and vertical bone grafting.

## 3. Results

The present narrative review seems to support the use of 3D-PFTM in conjunction with rhBMP-2 for the reconstruction of severely resorbed alveolar bone, both horizontally and vertically, with a focus on the following points:

- *Clinical efficacy:* the combination of rhBMP-2 and a 3D-preformed titanium mesh (3D-PFTM) proved effective for reconstructing severely resorbed alveolar bone;
- *Osteoinductive potential*: rhBMP-2 demonstrated strong osteoinductive activity, even in patients with compromised bone regenerative capacity;
- *Structural stability:* the use of 3D-PFTM, particularly the OssBuilder system, provided reliable structural support for bone regeneration;
- *Implant success*: the combined approach resulted in stable bone regeneration and long-term implant success;
- *Therapeutic value*: the so called BOXAM (BMP-2, Oss-builder, xenograft, allograft, maintenance) technique appears to be a promising and reproducible strategy in dental and maxillofacial surgery.

The following three clinical cases were treated with the BOXAM technique, in line with literature.

*Case 1: Vertical and Horizontal Bone Augmentation with Simultaneous Implant Placement in the Mandibular Anterior Region*

**Figure 4:**
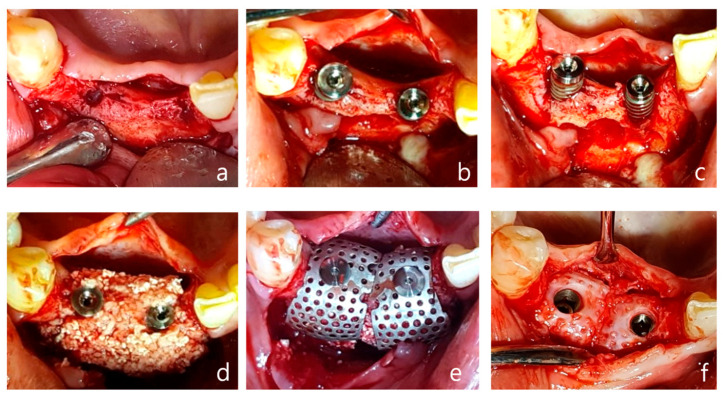

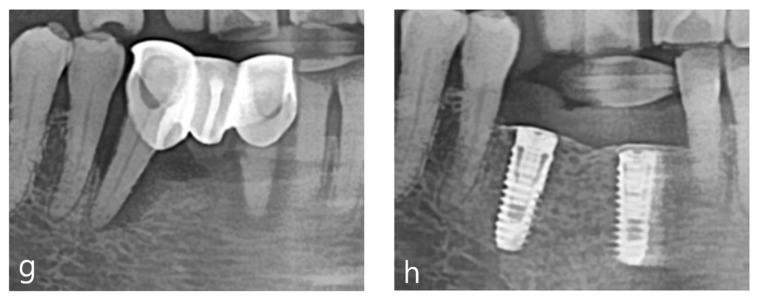
Case 1: vertical and horizontal bone augmentation with simultaneous implant placement in the mandibular anterior region. (**a**) Occlusal view after flap reflection following the incision at the surgical site. (**b**,**c**) After placing a 3.5 × 10 mm fixture in the central incisor and a 4.0 × 10 mm fixture in the canine, anchors were secured, indicating the need for vertical and horizontal bone augmentation. (**d**) Application of allograft and xenograft. (**e**) Application of horizontal-type Ossbuilder Ti-mesh and securing of the cover cap. (**f**) Three months post-operation, the Ti-mesh was removed during the second surgery, revealing appropriately newly formed alveolar bone around the fixtures. (**g**) Pre-operative panoramic radiograph. (**h**) Post-operative panoramic radiograph.

A 68-year-old healthy female patient presented to Wonju Dental Clinic for implant treatment. Due to significant alveolar bone resorption and severe mobility of the existing bridge from the right mandibular central incisor to the canine, extraction was planned (Figure 4a–g). Implant surgery was performed three months after the extractions.

Using a surgical scalpel (15C), a mid-crestal incision was made in the keratinized gingiva along the crest of the alveolar ridge at the edentulous site extending from the right mandibular central incisor to the canine, with vertical incisions being made at the adjacent teeth (Figure 4a).

As reported in Figure 4, a full-thickness flap was then elevated. Drilling was performed at the implant placement site, followed by a periosteal release incision on the buccal flap. After the fixture was placed, an appropriate anchor was secured to the top of the implant (Figure 4b,c). Allograft and xenograft materials saturated with rhBMP-2 were prepared, with the allograft (Regenoss^®^, NeoBiotec, Seoul, Republic of Korea) being layered around the fixture and the xenograft (A-Oss^®^, Osstem, Republic of Korea) being layered externally and matching the anchor height(Figure 4d). Two horizontal-type meshes were selected for vertical and horizontal bone augmentation, and the mesh was trimmed to fit the defect while ensuring complete coverage of the graft material and avoiding contact with adjacent teeth. The prepared mesh was placed over the anchor and secured with a cover cap (Figure 4e). The flap was sutured using interrupted sutures and vertical mattress sutures, and modified Laurell sutures were also employed. Approximately 4–5 mm of vertical bone augmentation was achieved. A secondary surgery was performed three months later to remove the mesh, which resulted in the attainment of excellent bone quality (Figure 4f–h). Five years post the operation, the bone and implant remain well-maintained without any complications.

*Case 2: Vertical and Horizontal Bone Augmentation with delayed Implant Placement in the Maxillary Anterior Region*

**Figure 5:**
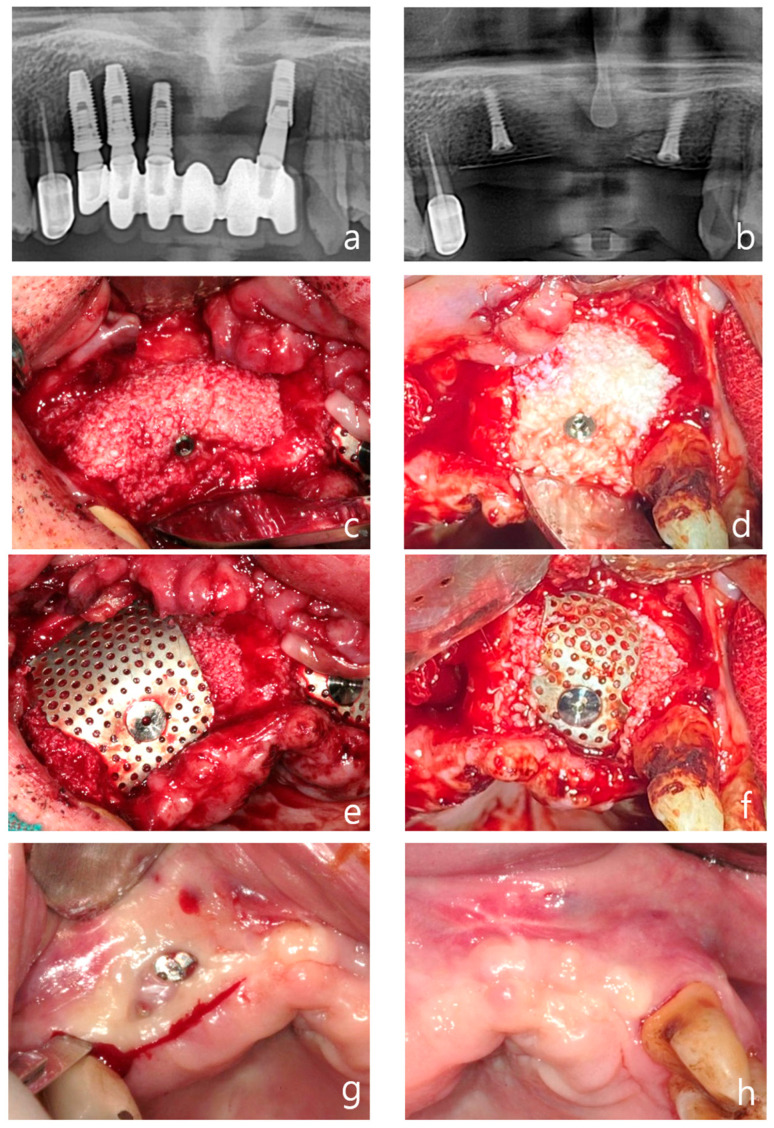

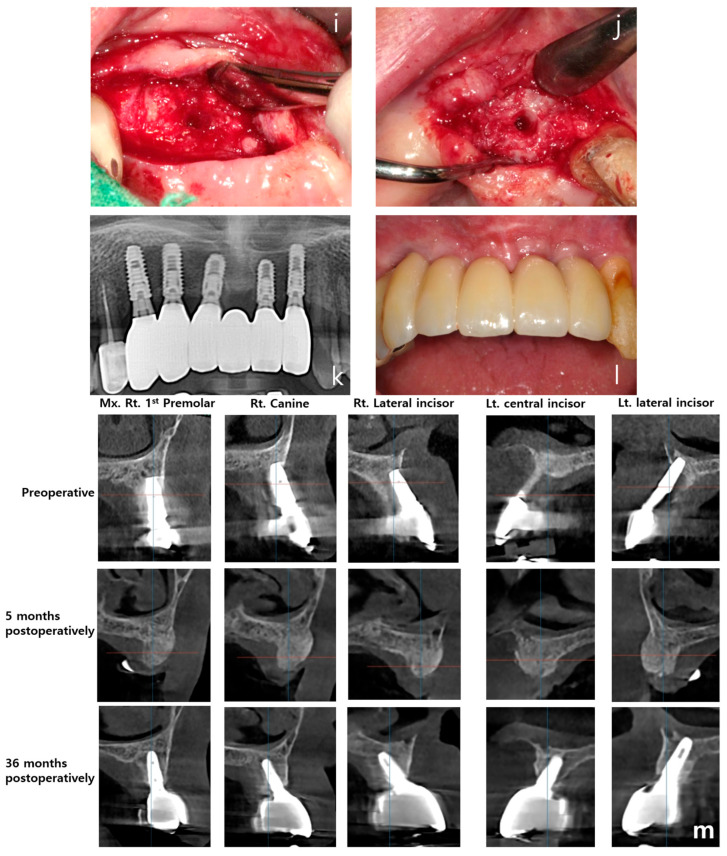
Case 2: vertical and horizontal bone augmentation with delayed implant placement in the maxillary anterior region. (**a**) Pre-operative panoramic radiograph. (**b**) Post-operative panoramic radiograph. (**c**,**d**) Following the placement of two 11.5 mm tenting screws in the right maxillary canine area and between the left maxillary central and lateral incisors, allograft and xenograft were applied for vertical and horizontal bone augmentation. (**e**,**f**) Application of vertical-type Ossbuilder Ti-mesh and securing of the cover cap. (**g**,**h**) Intraoral view at three months post-surgery. Although exposed cover caps were observed, high-quality alveolar bone formation was noted around the tenting screws during the second surgery. (**i**) Intraoral view of the left surgical site at four months post-operation. (**j**) At four months post-surgery, excellent alveolar bone regeneration was observed during the second surgery. (**k**) Panoramic radiograph after placement of the final prosthesis. (**l**) Intraoral view at 36 months post-operation. (**m**) Pre-operative, 5-month post-operative, and 36-month post-operative CT images (sagittal view) for each implant site. The vertically augmented bone graft material is observed to be well-maintained and remodeled.

A 68-year-old healthy male patient presented to Wonju Dental Clinic for implant treatment in the maxillary anterior region. Severe alveolar bone loss and gingival recession due to peri-implantitis were observed from the right maxillary first premolar to the left maxillary lateral incisor, which necessitated the removal of the affected fixtures (Figure 5a).

A mid-crestal incision and vertical incisions at the adjacent teeth were made to elevate a full-thickness flap on both the buccal and palatal sides. The condition of the soft tissue was not optimal for the simultaneous placement of new fixtures after the removal of the existing ones. Therefore, 11.5 mm tenting screws were placed at the site of the right maxillary canine and between the left maxillary central and lateral incisors (Figure 5b). An allograft (SureOss^®^, HansBiomed, Seoul, Republic of Korea) saturated with rhBMP-2 was placed around the screws, with a xenograft (THE Graft^®^, Purgo, Seongnam, Republic of Korea) saturated with rhBMP-2 being layered externally (Figure 5c,d). Vertical bone augmentation was performed using vertical-type mesh (Figure 5e,f). Approximately 8–9 mm of vertical bone augmentation was achieved

Two months post-operation, the cover screw on the right maxillary site became exposed. The patient was prescribed chlorhexidine and oral probiotics for daily use, and was advised to visit the dental clinic regularly for local disinfection of the area. Fortunately, no further infections occurred, and at three months post-operation, a secondary surgery was performed to remove the tenting screw and mesh from the left maxillary site (Figure 5g). Although the cover screw was exposed and the secondary surgery was conducted relatively early, at three months post-surgery, the bone quality was found to be excellent (Figure 5h,i).

One month later, at four months post-surgery, a secondary procedure was performed on the left maxillary site, which also exhibited excellent bone quality (Figure 5h–j). The patient preferred not to have the secondary surgery and implant placement performed simultaneously. Therefore, five months after the bone grafting, implants were placed in the left maxillary first premolar, canine, lateral incisor, and central incisor sites using a surgical guide.

A follow-up panoramic radiograph taken after the final prosthesis delivery showed satisfactory implant positioning and prosthetic outcome (Figure 5k). At 36 months post-operatively, the intraoral view demonstrated long-term stability of the soft tissue and prosthetic structure (Figure 5l). Finally, sagittal CT images taken pre-operatively, at five months, and at 36 months post-operatively illustrated the maintenance and remodeling of the vertically augmented graft material at each implant site (Figure 5m).

Now, three years and six months after the vertical bone augmentation, both the bone and implants remain stable without any complications.

*Case 3: Vertical & Horizontal Bone Augmentation with Implant Placement in the Mandibular Posterior Region of a Patient with a History of MRONJ*

**Figure 6:**
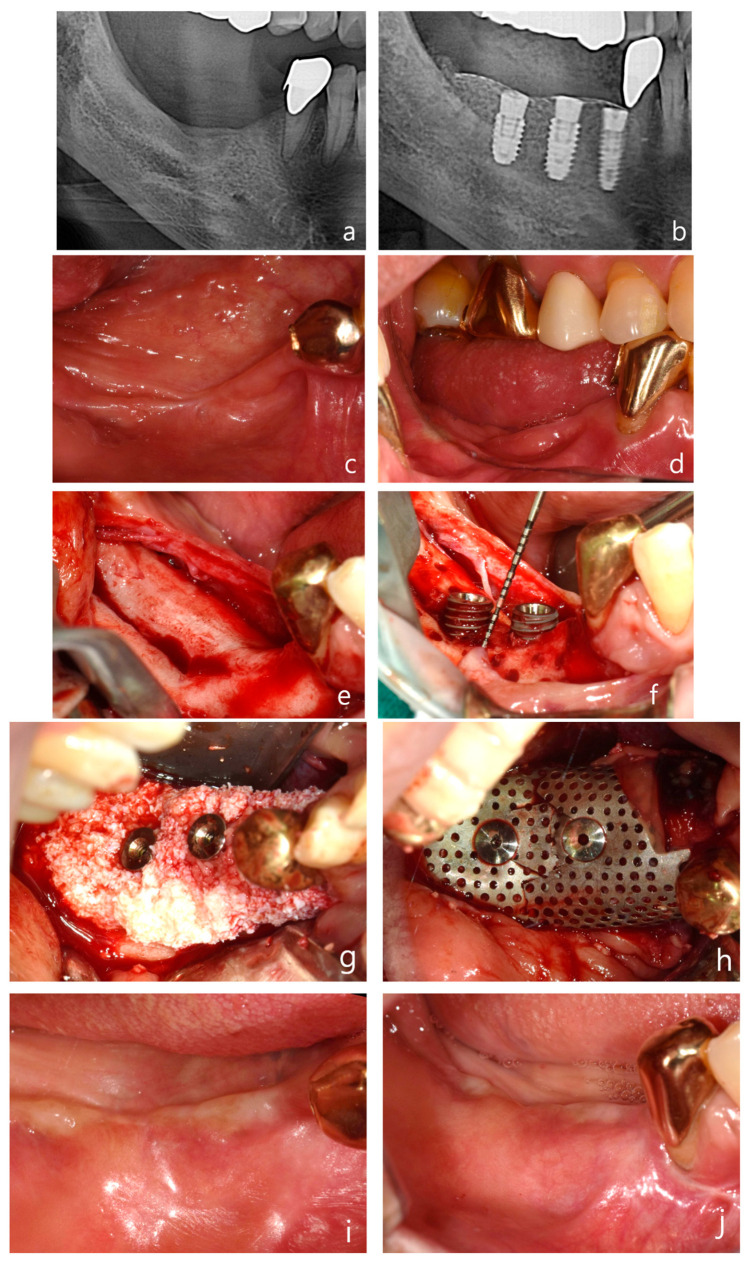

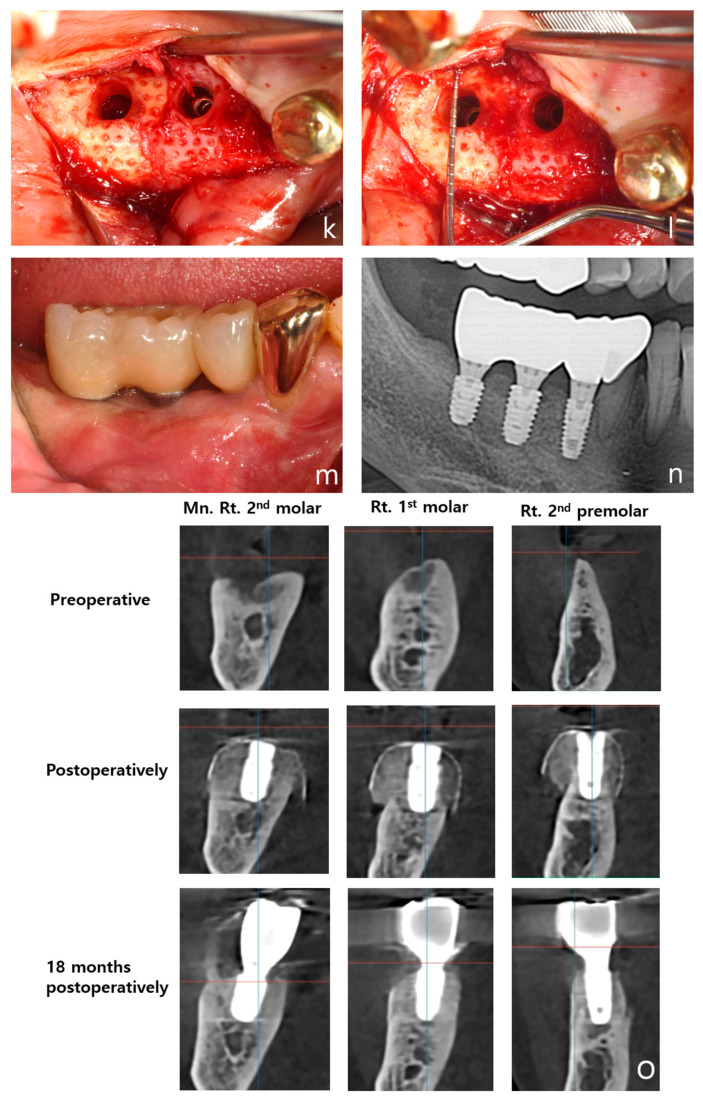
Case 3: Vertical and horizontal bone augmentation with implant placement in the mandibular posterior region of a patient with a history of MRONJ. (**a**) Pre-operative panoramic radiograph. (**b**) Post-operative panoramic radiograph. (**c**,**d**) Pre-operative intraoral photograph of the surgical site. (**e**) Severe vertical alveolar bone deficiency was observed after flap reflection following the incision at the surgical site. (**f**) Approximately 5 mm of thread exposure was observed on the fixture of the first molar (5.0 × 8.5 mm) and the second molar (5.0 × 7 mm). (**g**) After securing a 3 mm anchor, allograft and xenograft were layered for bone augmentation. (**h**) Application of vertical-type Ossbuilder Ti-mesh and securing of the cover cap. (**i**,**j**) Intraoral view at 4 months post-surgery. (**k**,**l**) High-quality alveolar bone formation was noted around the fixtures during the second surgery. (**m**) Intraoral view at 18 months post-operation. (**n**) Panoramic radiograph after placement of the final prosthesis. (**o**) Pre-operative, post-operative, and 18-month post-operative CT images (sagittal view) for each implant site. The vertically augmented bone graft material is observed to be well-maintained and remodeled.

The patient, a 63-year-old female with osteoporosis, presented with significant vertical alveolar bone resorption and tooth loss in the right mandibular second premolar and first and second molar regions, which necessitated implant placement (Figure 6a–d). The patient had a history of osteoporosis and had previously developed MRONJ (medication-related osteonecrosis of the jaw) as a result of osteoporosis medication, for which they had received treatment.

A mid-crestal incision was made in the keratinized gingiva of the edentulous area using a surgical scalpel (15C), with the distal extension of the crestal incision terminating within 2 mm of the retromolar pad. For surgical access, a distal oblique vertical incision was made toward the coronoid process of the mandible. A mesial vertical incision was made at the adjacent tooth, as well as a horizontal incision that extended 5 mm below the gingival margin on the lingual side of the adjacent teeth (Figure 6e).

The buccal flap was mobilized by performing a periosteal release incision with a surgical scalpel (15C), while the lingual flap was similarly mobilized using a periosteal elevator. After confirming that both flaps were adequately released to the desired height, drilling was performed at the implant placement sites using a pre-prepared surgical guide. To promote angiogenesis in the recipient bone bed, the cortical bone was perforated using a 1 mm diameter tenting screw drill from the Ossbuilder Kit^®^ (Osstem, Seoul, Republic of Korea). Fixtures were placed in the second premolar and first and second molar sites, and 3 mm anchors were secured to each fixture (Figure 6f).

The mesh was positioned and adjusted to fit the defect before the placement of the bone graft material. In this case, two vertical-type Ossbuilder meshes with a buccal width of 20 mm were utilized. One mesh was positioned at the second molar to cover the bone graft from the central implant site to 10 mm posterior to the implant site, while the other mesh was placed at the first molar to cover the distal aspect of the second premolar. The meshes were carefully trimmed to ensure that the cover caps did not interfere with each other between the first and second molar sites.

Using a Minnesota retractor, the lingual flap was held back to prevent the bone graft material from spilling over to the lingual side during placement. The allograft, hydrated with rhBMP-2 (MyBone^®^, MSBIO, Busan, Republic of Korea) was applied around the fixtures, with the xenograft (A-Oss^®^, Osstem, Republic of Korea) being layered externally (Figure 6g). The prepared meshes were then positioned and secured with cover caps, which was followed by suturing (Figure 6h). In the first and second molar regions, approximately 8 mm of vertical augmentation was achieved, including a 5 mm vertical exposure of the fixtures and 3 mm of anchor height.

A secondary surgery was performed at 17 weeks post-operation, which revealed excellent bone quality (Figure 6i–l). The patient has now been followed for 18 months post-operation, with both the bone and implants remaining stable and without complications (Figure 6m). A panoramic radiograph taken after final prosthesis delivery confirmed successful functional and esthetic outcomes (Figure 6n). Sagittal CT images captured at pre-operative, post-operative, and 18-month follow-up stages illustrated the long-term maintenance and remodeling of the vertically augmented graft material (Figure 6o). The patient is currently taking parathyroid hormone therapy to treat of osteoporosis.

These cases demonstrate the effectiveness of combining rhBMP-2 and preformed titanium mesh in reconstructing severely resorbed alveolar ridges. Successful new bone formation was achieved in all cases, ensuring the long-term stability of the implants.

## 4. Discussion

After tooth extraction, the resorption of vertical and horizontal alveolar ridges can pose significant challenges in positioning dental implants in the ideal location to meet functional and esthetic requirements. Although various guided bone regeneration (GBR) techniques have been developed to increase the horizontal width of the alveolar ridge, vertical ridge augmentation remains a complex and challenging task, irrespective of the approach or biomaterials used. The “Sausage technique”, introduced by Istvan Urban, has been reported to facilitate successful bone regeneration by securing resorbable collagen membranes or non-resorbable membranes, such as perforated polytetrafluoroethylene titanium-reinforced membranes, with titanium pins and directing a graft mixture of autogenous and xenografts in a 1:1 ratio toward the ridge. In Urban’s sausage technique, a secondary surgery is performed nine months after bone grafting to remove the fixation pins and non-resorbable membrane, which is followed by implant placement [24,25].

Alessandro Cucchi and co-workers in 2021 reported a similar approach, combining autogenous grafts and xenografts in a 1:1 ratio with a customized CAD/CAM titanium mesh to achieve both horizontal and vertical bone augmentation prior to implant placement [26]. Their method also utilized fixation mini screws, and they reported that both the use of the mesh alone and the mesh covered with a long-lasting membrane were effective. In Cucchi’s report, a secondary surgery was conducted six months after bone grafting to remove the fixation mini screws and titanium mesh, which was followed by implant placement [26].

In this study, the BOXAM technique has been developed, which utilizes rhBMP-2 pre-mixed with allografts and xenografts for horizontal and vertical alveolar ridge augmentation. The technique involves layering allografts, which are favorable for bone remodeling, around fixtures or tenting screws, while placing xenografts, which excel in volume maintenance, on the outer side. The procedure employs the 3D-PFTM Ossbuilder, and the acronym “BOXAM” is derived from the first letters of BMP, Ossbuilder, allograft, xenograft, and maintenance. The advantage of the BOXAM technique is that the anchors and cover caps of the Ossbuilder can be manually secured, which makes it feasible to use when an initial stability of over 10 N is achieved during implant placement. This allows for simultaneous vertical ridge augmentation during implant placement, and the use of rhBMP-2 accelerates the ossification of the graft material, potentially reducing the treatment time compared to traditional vertical ridge augmentation methods. Indeed, using the BOXAM technique, secondary surgery was performed just four months after achieving 4–5 mm of vertical bone augmentation, and resulted in excellent bone quality and implant stability, as well as a timeframe notably shorter than the 9-month or 6-month periods reported by Urban and Cucchi, respectively. The use of this technique also minimizes post-operative pain and discomfort for the patient, as it eliminates the need for harvesting autogenous bone. Another advantage of the BOXAM technique is that it is less invasive and simpler compared to other GBR methods that require fixation screws, as the absence of such screws makes the secondary surgery for membrane removal less complex. However, a limitation of the BOXAM technique is that it cannot be used when the distance to the inferior alveolar nerve is too short to allow the placement of fixtures or tenting screws.

The combination of rhBMP-2 and 3D-PFTM with allografts and xenografts (BOXAM technique) has emerged as a critical therapeutic strategy in alveolar bone reconstruction, ensuring long-term success in dental implant surgery. RhBMP-2 has strong osteoinductive properties that stimulate the proliferation, migration, and differentiation of mesenchymal stem cells into osteoblasts and plays a role in regulating the expression of target genes involved in bone physiology, being particularly effective in regenerating severely resorbed alveolar bone [27].

3D-PFTM enhances the effectiveness of rhBMP-2 by providing structural stability and creating an optimal environment for bone regeneration. The non-resorbable nature of titanium mesh ensures that it maintains its structural integrity over an extended period, which makes it particularly effective in complex bone defects. Titanium, known for its high biocompatibility, minimizes inflammatory reactions and the risk of rejection, which is crucial for the long-term success of dental implants [21,22,23].

Despite the commercialization and clinical application of BMPs, they are currently available only in liquid form. This presents challenges, as BMPs alone are difficult to handle, have low stability, and are prone to rapid diffusion, absorption, and degradation within the body, which makes effective bone formation induction challenging [28].

Therefore, one of the most critical factors in the clinical application of rhBMP-2 is the selection of an appropriate carrier. An ideal carrier should promote an optimal inflammatory response, be fully biodegradable, and provide sufficient porosity to support cell infiltration, proliferation, and the formation of new blood vessels at the site of bone regeneration. Furthermore, it must protect BMPs from degradation while preserving their bioactivity, which enables a controlled and sustained release to effectively promote bone formation at the defect site. Additionally, the carrier should be easily sterilized, user-friendly, stable during storage, and commercially viable for large-scale production [29].

Several carriers, including autografts, allografts, xenografts, synthetic bone grafts, demineralized dentin matrix (DDM), and collagen, are commonly used in conjunction with rhBMP-2 [30,31,32]. Since rhBMP-2 is typically stored in liquid form, an absorbable collagen sponge (ACS) is often used as the carrier. However, due to the lack of structural stability in collagen carriers, titanium mesh is frequently employed in conjunction with rhBMP-2/ACS for horizontal or vertical bone augmentation to prevent the collapse of the osteogenic space under pressure [33].

In this study, various commercially available allografts and xenografts were utilized as carriers, all of which yielded excellent results. Kloss et al. reported that the combination of a bovine bone substitute material with allogeneic bone granules provides stable alveolar ridge augmentation, effectively maintaining the bone volume and tissue support over a three-year period without the need for autogenous bone harvesting [34]. Seo et al. reported that allografts, when used as a carrier for rhBMP-2, significantly enhance bone regeneration compared to other carriers, such as absorbable collagen sponges. This finding underscores the effectiveness of allografts as a superior carrier option for promoting bone regeneration in maxillofacial applications [30].

Allogeneic bone substitutes, which come from human donors, are carefully processed and sterilized to ensure that they are safe and to minimize immune reactions [35]. They are widely available, which makes them a convenient choice for dental procedures even if several techniques has been developed to only use collagen, autologous bone, or a prosthetic socket preservation (PSP) rather than a bone substitute, and their use seems promising in term of success rate and long follow-up [36,37,38].

These substitutes support bone growth by providing a structure that promotes quick natural bone regeneration [39]. Studies have shown that using allografts for horizontal ridge augmentation is just as effective as using autogenous bone, with no major differences in implant success, bone resorption, or complication rates [40,41].

Xenogeneic bone substitutes, usually derived from animals like cows or pigs, are also processed to reduce the risk of disease and immune reactions [42]. These substitutes are commonly used in dental implants and are known for their excellent ability to maintain the bone volume and to be a scaffold for bone growth. Bovine bone substitutes treated at high temperatures are particularly good at maintaining the bone volume over time due to their slow resorption rate [43].

In clinical practice, when using rhBMP-2 and titanium mesh with an allograft alone, the initial bone volume appears satisfactory at the time of the second surgery following bone grafting. However, over time, there is a noticeable decrease in the volume of the grafted bone. Further, when a xenograft is layered over the allograft, it has been observed that the bone volume is better maintained over the long term. Therefore, in these cases from this study, allograft, known for its superior bone regeneration, was grafted around the fixture, while a xenograft was applied to the outer layer to help maintain bone volume.

The use of rhBMP-2 in the treatment of medication-related osteonecrosis of the jaw (MRONJ) appears to be a promising approach, based on the findings from several studies. RhBMP-2 has been shown to significantly enhance cell viability and upregulate RANKL (receptor activator of NF-κB ligand) and M-CSF (macrophage colony-stimulating factor) expression in bisphosphonate-treated osteoblasts, which suggests its potential to counteract the bisphosphonate-induced inhibition of bone remodeling. Kwon et al. reported the therapeutic use of rhBMP-2 in treating bisphosphonate-related osteonecrosis of the jaw (BRONJ), as it promotes osteoblast function and bone regeneration [44].

The application of rhBMP-2 combined with an absorbable collagen sponge has shown positive regenerative effects in MRONJ patients, particularly following sequestrectomy. The studies indicate that rhBMP-2 contributes to new bone formation by enhancing bone remodeling, a process that is often impaired in patients with MRONJ due to the inhibitory effects of bisphosphonates on osteoclast activity [45]. Additionally, a case series review reported successful mandibular reconstruction and rehabilitation in MRONJ patients with large bone defects using rhBMP-2/ACS and a surgical miniplate, without the need for additional grafting materials [46].

These reports indicate that rhBMP-2 may have a positive effect on bone remodeling and cell viability, supporting its potential in the treatment of MRONJ patients. While further clinical research is necessary, it is believed that rhBMP-2 could help prevent the occurrence of MRONJ and improve bone graft outcomes during implant placement and alveolar ridge reconstruction in patients at risk of MRONJ due to their current medication or in those with a history of MRONJ where recurrence is a concern.

In implant ridge augmentation, membranes are crucial and can be classified into absorbable and non-absorbable types, each of which offers distinct advantages and limitations. Resorbable membranes gradually degrade over time, which eliminates the need for a second surgery to remove them and thus reduces the patient’s surgical burden and minimizes post-operative complications [20].

This makes them an effective treatment option, particularly for simpler bone defects. They are also relatively cost-effective and can be applied in various clinical scenarios. However, their lower physical strength may limit long-term structural support, potentially leading to suboptimal bone regeneration outcomes [47]. Resorbable membranes may also degrade before bone regeneration is complete, which could negatively affect the final outcome, particularly in complex cases.

Non-resorbable membranes such as polytetrafluoroethylene (PTFE) and titanium mesh, on the other hand, provide robust structural support, which makes them ideal for complex bone defects. Although non-resorbable membranes are associated with complications and the need for secondary surgery, their space-maintaining capability combined with volume stability, particularly in titanium-reinforced materials, remains clinically irreplaceable, especially in cases of large, non-contained bone defects or vertical augmentations [48,49,50].

Although expanded polytetrafluoroethylene(e-PTFE) and high-density polytetrafluoroethylene (d-PTFE) membranes provide adequate barrier function and space maintenance, they are prone to deformation under pressure in cases of large vertical bone defects, which can negatively impact bone regeneration [51].

Several studies that included titanium-reinforced d-PTFE concluded that the titanium-reinforced d-PTFE membrane could more effective than the control membrane in vertical bone regeneration, as the porosity of the d-PTFE membrane is less than 0.3 microns and it could be less colonized by bacteria as a result [52].

To address this deficiency, titanium-reinforced membranes have been developed by integrating a titanium skeleton into the PTFE membrane, which enhances plasticity and volume stability, while also providing superior mechanical support and facilitating easy clinical placement under the flap [53,54].

Among non-resorbable membranes, titanium mesh is widely recognized for its structural integrity and biocompatibility [14]. Titanium mesh provides strong support, protecting the bone graft and guiding stable bone regeneration. This strength is particularly beneficial in complex bone defects, where maintaining the graft material’s position is crucial. Titanium’s biocompatibility minimizes inflammatory and rejection responses, contributing to the long-term success of dental implants [55].

Preformed titanium mesh offers several distinct advantages over standard titanium mesh. Preformed meshes are custom fabricated to fit specific anatomical sites, which reduces the need for intraoperative modifications and thus shortens the surgery time and improves accuracy. This ease of use enhances the clinician’s ability to predict and control surgical outcomes. Preformed meshes are available in various sizes and shapes, which makes them versatile tools for addressing different types of bone defects. This versatility is particularly valuable in complex reconstructions, where precise fitting of the mesh is essential for success [14]. Despite the higher cost and the potential need for additional customization, the benefits of preformed titanium mesh in terms of reduced surgery time and improved fit often justify the investment.

Although resorbable membranes seem to be promising in terms of morbidity and complications, in terms of the timing of resorption, some types of collagen membranes may also have effective results in terms of keeping the volume of bone formation, such as high-density dermal matrix or nono-structred collagen membrane, which can be used for bone grafting techniques [56,57,58]. The limitations of this study were related to the narrative review, due to the heterogeneity of the involved studies and the small sample of cases.

Neverless, OssBuilder^®^, a representative product of preformed titanium mesh, offers numerous advantages in clinical applications. This study highlighted that OssBuilder^®^ is available in multiple shapes and sizes, which makes it suitable for a wide range of bone defects. Its design allows for effective use in both vertical and horizontal ridge augmentations, providing flexibility in various clinical scenarios. Furthermore, OssBuilder’s anchors are available in different heights, which allows clinicians to select the appropriate size based on the implant depth and position, and to thereby reduce the risk of complications. In cases where there is no existing alveolar bone above the implant fixture, such as in vertical bone augmentation procedures, using traditional titanium mesh may result in the mesh being positioned directly above the implant fixture. This can lead to the insufficient formation of hard and soft tissues above the fixture, potentially increasing the risk of peri-implantitis in the long term. On the other hand, by using Ossbuilder^®^, one can select an anchor with a height of up to 3 mm, which increases the volume of hard and soft tissues above the implant and promotes long-term biological stability. Furthermore, Ossbuilder’s preformed design eliminates the need for additional intraoperative shaping, reducing the surgery time and enhancing procedural efficiency. Ossbuilder^®^ is pre-shaped with a curved surface, which eliminates the creation of angular or sharp edges that can occur when bending or cutting traditional titanium mesh. This design reduces irritation to the surrounding soft tissues after suturing. Moreover, because Ossbuilder^®^ can be directly connected and secured to the implant fixture or tenting screws, there is no need for additional screws for fixation. As a result, during the second surgery, the Ossbuilder^®^ can be removed with minimal incision, which makes it less invasive and much easier to handle compared to the removal of traditional non-resorbable membranes or titanium mesh, which requires the removal of fixation screws. Future directions of these evaluations should evaluate the possibility to customize titanium mesh or to use entirely late-resorbable membranes and materials.

## 5. Conclusions

This study confirms the clinical effectiveness of combining recombinant human bone morphogenetic protein-2 (rhBMP-2) with three-dimensional preformed titanium mesh (3D-PFTM) for the reconstruction of severely resorbed alveolar ridges. The strong osteoinductive capacity of rhBMP-2, even in patients with compromised bone regenerative potential, along with the structural support offered by 3D-PFTM—particularly the OssBuilder system—significantly contributes to successful bone regeneration and long-term implant stability. OssBuilder demonstrates several clinical advantages for guided bone regeneration. It is available in various shapes and sizes, which makes it adaptable for both vertical and horizontal ridge augmentation procedures. The system includes anchors of varying heights, which can be selected based on the implant depth and positioning to minimize complications. This technique was named the “BOXAM” technique, and it represents a promising therapeutic modality in the field of dental and maxillofacial surgery.

## Data Availability

No new data were created.

## Abbreviation

BOXAM: BMP-2, Oss-builder, xenograft, allograft, maintenance. rhBMP-2: recombinant human bone morphogenetic protein-2. BMP: bone morphogenetic protein. TGF-β: transforming growth factor-beta. 3D-PFTM: three-dimensional preformed Ti mesh. GBR: guided bone regeneration. PTFE: polytetrafluoroethylene. e-PTFE: expanded polytetrafluoroethylene. d-PTFE: high-density polytetrafluoroethylene. TCP/HA: tricalcium phosphate/hydroxyapatite. OB: OssBuilder. MRONJ: medication-related osteonecrosis of the jaw. DDM: demineralized dentin matrix. PSP: prosthetic socket preservation. RANKL: receptor activator of NF-κB ligand. M-CSF: macrophage colony-stimulating factor. ACS: absorbable collagen sponge.
